# Influence of Drying Methods on Phytochemical Retention of *Moringa oleifera* Leaves and Green Synthesis of Zinc Oxide Nanoparticles Using the Optimal *Moringa oleifera* Leaf Extract

**DOI:** 10.1002/fsn3.72443

**Published:** 2026-10-29

**Authors:** Tamanna Rahman, Fahima Siddikey, Md. Hassan Bin Nabi, Md Abu Rayhan, Wahidu Zzaman

**Affiliations:** ^1^ Department of Food Engineering and Tea Technology Shahjalal University of Science and Technology Sylhet Bangladesh

**Keywords:** antioxidant and antimicrobial activity, drying strategies, green synthesis, *Moringa oleifera*, storage stability, ZnO nanoparticles

## Abstract

Green synthesis of zinc oxide nanoparticles (ZnO‐NPs) using plant extracts is an environmentally friendly approach; however, the influence of leaf drying pretreatment on nanoparticle synthesis remains underexplored. This study first evaluated the effects of air‐, sun‐, oven‐, and freeze‐drying of *Moringa oleifera* leaves on phytochemical retention, and then used the extract obtained from the best‐performing drying treatment for the green synthesis of ZnO‐NPs. Freeze‐dried leaves retained the highest levels of phenolics, flavonoids, vitamin C, and β‐carotene and were therefore selected as the sole precursor for nanoparticle synthesis. GC–MS and HPLC confirmed the presence of bioactive compounds responsible for Zn^2+^ complexation and nanoparticle stabilization. The synthesized ZnO‐NPs exhibited a characteristic UV–Vis absorption peak at 365 nm with a band gap of 3.40 eV. FT‐IR, XRD, SEM, EDS, dynamic light scattering, and zeta potential analyses confirmed the formation of highly crystalline, flower‐like ZnO‐NPs with particle sizes of 45–82 nm (hydrodynamic diameter ~67 nm) and excellent colloidal stability (−32 mV). The biosynthesized ZnO‐NPs showed significantly higher antibacterial and antioxidant activities, faster radical scavenging kinetics, and excellent storage stability compared with analytical‐grade ZnO. These findings demonstrate that freeze‐drying is an effective pretreatment for producing stable and bioactive ZnO nanoparticles for food and biomedical applications.

## Graphical abstract

Fresh *Moringa oleifera* leaves were evaluated under different drying conditions to identify the treatment with superior retention of bioactive compounds. The selected freeze‐dried extract was used for green synthesis of ZnO nanoparticles, which exhibited characteristic physicochemical properties, concentration‐dependent antioxidant and antibacterial activities, and enhanced stability during storage.

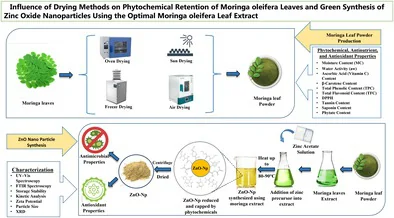

## 1 Introduction

Nanotechnology is an arising and fast‐evolving discipline offering novel applications across diverse sectors such as biomedicine, cosmetics, food packaging, agriculture, and environmental protection. In recent years, the demand for metal nanoparticle synthesis through conventional (physical and chemical) and green synthesis techniques has been steadily rising (Awlqadr et al. 2025). Among these methods, green synthesis has been recognized as a preferred alternative, using biological agents like plants, bacteria, fungi, and algae (Álvarez‐Chimal and Arenas‐Alatorre 2023; Bhalla et al. 2023; Rahman et al. 2026; Vanga and Satla 2025). It provides multiple benefits, notably minimal toxicity and economic feasibility, environmental compatibility, and the ability to fine‐tune particle size and shape via naturally occurring biomolecules. Plant‐based nanoparticle synthesis is gaining attention mainly because plant extracts contain various beneficial compounds that significantly influence the favorable physicochemical traits of nanoparticles (Bhalla et al. 2023). Current research highlights the significance of nanoparticles derived from metals as well as their oxides, including gold, copper, nickel, silver, and zinc synthesized through green methods, which offer environmentally friendly alternatives for diverse applications (Singh et al. 2021; Suresh et al. 2018; Yasmin et al. 2023). Due to their nontoxicity and biocompatibility, metallic nanoparticles are employed in the biomedical field for targeted therapeutic delivery (Cai et al. 2016), anticancer (Mishra et al. 2017), and antimicrobial treatments, as well as in food processing to improve flow, flavor, stability, color, and shelf life (Chadha et al. 2022; Akter et al. 2026).

Among the diverse nanomaterials studied, zinc oxide nanoparticles (ZnO‐NPs) are widely explored for their unique physicochemical properties (Suresh et al. 2018). These characteristics encompass semiconductor properties, ultraviolet light absorption, and strong antibacterial as well as antioxidant effects (Shashikala et al. 2024). Moreover, the U.S. Food and Drug Administration (FDA) recognizes zinc oxide (ZnO) as a GRAS substance, making ZnO nanoparticles promising candidates for health and wellness applications, particularly in combating microbial infections, oxidative stress, and inflammation (Espitia et al. 2025).

*Moringa oleifera* , recognized as “miracle plant,” is renowned for its rich nutritional profile and abundance of phytochemical compounds encompassing polyphenols, flavonoids, terpenoids, tannins, and saponins (Arshad et al. 2025; Wilcaso et al. 2025). It is widely recommended for disease prevention due to its immune‐boosting properties and the presence of beneficial constituents, for instance, carbohydrates, proteins, fatty acids, functional peptides, and phenolic compounds. This unique composition makes moringa a promising candidate for various health‐promoting applications. Optimizing extraction methods, such as vacuum‐assisted extraction, can significantly enhance the yield and bioactivity of *M. oleifera* leaf compounds, supporting their use in functional foods and green nanoparticle synthesis (Espitia et al. 2025).

Drying is a widely employed technique to preserve plant materials, prolong shelf life, and facilitate further processing (Zhao et al. 2025). Studies show that different drying methods, such as freeze‐drying, air‐drying, and oven‐drying, significantly influence the retention of nutrients and bioactive compounds in leafy vegetables and medicinal plants, while co‐fermentation of *M. oleifera* leaves with Fuzhuan brick tea enhances phenolic content, antioxidant activity, and functional properties, highlighting the importance of optimized drying and pretreatment for functional applications (Ademiluyi et al. 2018; Li et al. 2023, 2024; Mahmud et al. 2026). Freeze drying, or lyophilization, uses low temperature and vacuum to sublimate ice, minimizing structural damage and preserving thermolabile compounds. Air drying, a traditional method, relies on ambient air to gradually remove moisture and is simple and cost‐effective, though prolonged drying can degrade sensitive compounds (Jurčević Šangut et al. 2024). Sun drying exploits solar energy to dehydrate materials, offering a low‐cost approach, yet exposure to fluctuating temperatures and direct sunlight may lead to losses of volatile and bioactive compounds. Oven drying applies controlled heat to accelerate moisture removal, ensuring uniform drying but potentially compromising heat‐sensitive nutrients and antioxidants (Zhao et al. 2025). However, compared to chemical approaches, green production of metal nanoparticles offers an environmentally sound alternative; the effect of preprocessing methods, such as drying for *M. oleifera* leaves, on nanoparticle bioactivity remains underexplored, which optimizes retention of nutritional and functional components.

Despite numerous reports on the green synthesis of ZnO nanoparticles using *M. oleifera* extracts, the influence of drying pretreatment on phytochemical retention and its subsequent impact on nanoparticle formation and biological performance remains largely unexplored. Specifically, it is still unclear which drying method best preserves the bioactive constituents responsible for nanoparticle reduction, capping, and stabilization, and how these changes affect the antioxidant and antibacterial properties of the synthesized ZnO‐NPs. Therefore, this study systematically compared freeze‐, air‐, sun‐, and oven‐drying of *M. oleifera* leaves to identify the pretreatment that best preserves phytochemicals, and subsequently used only the optimal (freeze‐dried) extract for the green synthesis and comprehensive characterization of ZnO nanoparticles. The novelty of this work lies in establishing, through a two‐stage experimental design, (i) the relationship between drying pretreatment and phytochemical retention in *M. oleifera* leaves, and (ii) the functional performance of ZnO nanoparticles subsequently synthesized from the best‐performing (freeze‐dried) extract, thereby providing practical insights for selecting appropriate pretreatment strategies in plant‐mediated nanoparticle production.

## 2 Methodology

### 2.1 Preparation of Moringa Leaf Powders by Diverse Drying Strategies

Fresh *M. oleifera* Lam. leaves (approximately 2.5 kg, corresponding to leaves harvested from 15 to 20 mature trees) were collected from Bangladesh Institute of Research & Training on Applied Nutrition (BIRTAN), Araihazar, Narayanganj, Bangladesh (23.635° N, 90.687° E). Mature, disease‐free leaves were selected and cleaned under running tap water.

Fresh *M. oleifera* leaves exhibited an initial moisture content of 76.8% ± 0.5% (wet basis) using the AOAC oven‐drying method (AOAC 2019). After washing and draining, the leaves were divided into four equal portions for different drying treatments. Freeze‐drying was carried out at −50°C under a vacuum of < 0.1 mbar for 48 h. Air‐drying was performed at 25°C ± 2°C under 60% ± 5% relative humidity for 72 h. Sun‐drying was conducted under natural sunlight (30°C–35°C, 55%–70% relative humidity) for 24 h with adequate ventilation and periodic turning of the leaves. Oven‐drying was performed at 40°C for 18 h in a forced‐air hot‐air oven. Drying continued until constant weight was achieved. The final moisture contents of the freeze‐, air‐, sun‐, and oven‐dried samples were 3.48% ± 0.02%, 4.76% ± 0.03%, 5.42% ± 0.02%, and 5.94% ± 0.03%, respectively. After drying, all samples were ground using the same laboratory grinder and passed through a 60‐mesh sieve (approximately 250 μm) to obtain a uniform particle size prior to extraction and subsequent analyses.

### 2.2 Phytochemical, Antinutrient, and Antioxidant Properties of *M. oleifera* Leaf Powder

#### 2.2.1 Moisture Content (MC)

MC was determined using the oven‐drying method. One gram of material was heated at 105°C until a constant mass was reached. MC was evaluated from the reduction in mass during drying (Nielsen 2017).

#### 2.2.2 Water Activity (a_w_)

Water activity of DML powder, at 25°C, was determined with a WA‐60A water activity meter (USA) having 0.001 a_w_ sensitivity, after reaching equilibrium (Islam et al. 2024).

#### 2.2.3 Estimation of Ascorbic Acid (Vitamin C)

Vitamin C in dried samples was quantified following Vallespir et al. (2019) with minimal adjustments. 1 g powder was extracted with 10 mL of sodium oxalate (0.056 M), settled for 5 min, filtered, and 0.5 mL of the filtrate was made up to volume to 5.0 mL with the same solution. Absorbance was assessed at 266 nm using a UV–vis spectrophotometer (T60U, PG Instruments Ltd., UK), with sodium oxalate as the blank. Quantification was determined according to a calibration curve for L‐ascorbic acid references.

(Absorbance = 0.0035 × concentration + 0.015; correlation coefficient, *R* ^2^ = 0.996).

#### 2.2.4 Estimation of β‐Carotene

For β‐carotene quantification, 10 mL of distilled water was used to homogenize the samples, then hexane (5 mL) was added for carotenoid extraction. Solution stood undisturbed for 5 min before being shaken once more to promote phase separation. The hexane layer, containing the carotenoids, was then measured spectrophotometrically at 452 nm (Islam et al. 2024). β‐Carotene concentration was determined from a standard calibration curve represented by the equation: *y* = 0.634*x* + 0.0107, with *R* ^2^ = 0.997.

#### 2.2.5 Estimation of Total Phenolic Content (TPC)

The TPC of moringa leaf powders was determined using the Folin–Ciocalteu method, with slight modifications based on Lamuela‐Raventós (2018). A 0.5 g powder was combined with 12 mL of 80% methanol and then centrifuged for 10 min at 5000 rpm. For the test, 0.5 mL extract was combined with 2.5 mL of 10% Folin–Ciocalteu reagent, and after 5 min, 2.0 mL of sodium carbonate (7.5%) was added. After 30 min of dark incubation, the mixture's absorbance at 765 nm was recorded. A standard curve was established with gallic acid as the reference compound, and outcomes were expressed in milligrams of gallic acid equivalents (mg GAE/g DW). TPC was measured via the calibration equation, *y* = 0.0004*x* + 0.0013, which has an *R* ^2^ value of 0.998.

#### 2.2.6 Determination of Total Flavonoid Content (TFC)

TFC was assessed by use of the colorimetric technique with aluminum chloride. 2.9 mL of distilled water, 1.7 mL of 95% ethanol, 0.1 mL of 10% aluminum chloride and also 1 M potassium acetate were mixed to create a 0.5 mL extract combination, which was then permitted to sit for 30 min. Then absorbance was determined at 415 nm. A blank solution was prepared by substituting aluminum chloride with distilled water in the same volume. Using a standard calibration curve, the flavonoid content was determined and is expressed as milligrams of quercetin equivalents per gram of dry weight (mg QE/g DW) (Chang et al. 2020; Gidamo 2023). The linear regression equation used was: *y* = 0.0038*x* + 0.0256, with an *R* ^2^ value of 0.9993. (Chang et al. 2020; Gidamo 2023). The linear regression equation used was: *y* = 0.0038*x* + 0.0256, with an *R* ^2^ value of 0.9993.

#### 2.2.7 DPPH Free Radical Assay

The DPPH test was used to assess the antioxidant properties of the samples in order to compare their efficacy with that of Saikia et al. (2015). 1.4 mL of DPPH (0.1 mM) in methanol was mixed to each 100 μL sample, and then kept for 30 min at ambient conditions away from light. The scavenging of DPPH radicals was identified by a color shift from violet to pale yellow. Ascorbic acid, prepared at the same concentration (100 μg/mL) served as the reference antioxidant and underwent the same treatment. Absorbance of all mixtures was recorded at 517 nm.

#### 2.2.8 Tannin Content Determination

The tannin content was evaluated following Ademiluyi et al. (2018), with minimal modification. Briefly, 0.25 g of the sample was placed into a 60 mL sample bottle, and before sealing 15 mL of 70% aqueous acetone was added. The mixture was centrifuged at 1000×*g* after being shaken for 2 h at 30°C. After being collected, the supernatant was kept on ice. A 0.2 mL aliquot of the solution was then combined with Folin–Ciocalteu reagent (0.5 mL) and 0.8 mL of distilled water; 1 mL of a 0.5 mg/mL tannic acid standard was then mixed with the same reagent. After being vortexed, the mixtures were kept undisturbed for 40 min, and at 725 nm absorbance was determined after 3 mL of Na_2_CO_3_ was added. Tannic acid equivalents were used to express the tannin content.

#### 2.2.9 Saponin Content Determination

The presence of saponin concentration was ascertained following Ademiluyi et al. (2018), with minor adjustments. In brief, in a beaker, 2.5 g of sample was mixed with 100 mL of isobutyl alcohol and stirred for 5 h. 40% saturated magnesium carbonate was used to clarify the extract, and in a volumetric flask (50 mL), clear solution (1 mL) was combined with 2 mL of 5% FeCl_3_ before being brought to volume with distilled water. The standard saponin solution was 0.2 mg/mL. After 30 min of settling, the absorbance at 380 nm was measured and the saponin content was computed using conventional saponin equivalents.

#### 2.2.10 Phytate Content Determination

Phytate levels were measured following Ademiluyi et al. (2018), with minor modifications: 4 g of powder were filtered through Whatman No. 1 paper after being steeped in 100 mL of 2% HCl for 3 h. As an indicator, 30 mL of the filtrate were mixed with 6 mL of 0.3% ammonium thiocyanate. The solution was titrated with iron (III) chloride solution containing 1.95 mg of iron per mL until a brownish‐yellow color lasted for 5 min after 53.5 mL of distilled water was added to correct the pH. The titration findings were used to compute the phytate concentration.

#### 2.2.11 GC–MS Analysis of Freeze‐Dried *M. oleifera* Leaf Extract

To identify the volatile and semi‐volatile phytochemical constituents present in the optimized freeze‐dried *M. oleifera* leaf extract used for ZnO nanoparticle synthesis, GC–MS analysis was performed on the same aqueous extract described in Section 2.3. The analysis was carried out using a Shimadzu GCMS‐QP2020 gas chromatograph coupled with a quadrupole mass spectrometer (Shimadzu Corporation, Kyoto, Japan). Chromatographic separation was achieved using a DB‐5MS fused‐silica capillary column (30 m × 0.25 mm i.d., 0.25 μm film thickness; 5% phenyl–95% dimethylpolysiloxane stationary phase). Helium (99.999% purity) was used as the carrier gas at a constant flow rate of 0.80 mL/min. A 1 μL aliquot of the prepared sample was injected in split mode at a split ratio of 1:40, with the injector temperature maintained at 250°C.

The GC oven temperature was initially maintained at 50°C for 2 min, followed by an increase to 250°C at 10°C min^−1^ and held for 2 min. The total chromatographic run time was approximately 22 min. Mass spectrometric detection was performed in electron‐impact (EI) ionization mode at 70 eV. The ion‐source, interface, and quadrupole temperatures were maintained at 230°C, 280°C, and 150°C, respectively. Data were acquired in full‐scan mode over an m/z range of 35–500.

The detected compounds were identified by comparing their experimental mass spectra with spectra in the NIST Mass Spectral Library (version 2.0). Linear retention indices (LRIs) were calculated using a homologous series of *n*‐alkanes (C_8_–C_30_) analyzed under identical chromatographic conditions and compared with published values. Major constituents were further confirmed using authentic analytical standards where available. The relative abundance of each constituent was calculated using the GC peak‐area normalization method and expressed as relative peak area (%). The GC–MS analysis was conducted specifically on the freeze‐dried *M. oleifera* leaf extract subsequently used for ZnO‐NP synthesis (Bhalla et al. 2023; Natrayan et al. 2024).

#### 2.2.12 HPLC Analysis of Phenolic and Flavonoid Compounds

The phenolic acids and flavonoid compounds in the optimized freeze‐dried *M. oleifera* leaf extract were analyzed using a high‐performance liquid chromatography (HPLC) system (Agilent 1260 Infinity II, Agilent Technologies, Santa Clara, CA, USA) equipped with a diode‐array detector (DAD). Chromatographic separation was achieved using a ZORBAX Eclipse Plus C18 column (250 × 4.6 mm i.d., 5 μm particle size). The mobile phase consisted of solvent A (0.1% formic acid in ultrapure water) and solvent B (acetonitrile). The gradient program was: 0–5 min, 5% B; 5–15 min, 15% B; 15–25 min, 25% B; 25–35 min, 35% B; 35–40 min, 50% B; 40–45 min, 70% B; 45–50 min, 5% B. The column temperature was maintained at 30°C, the flow rate was 1.0 mL/min, and the injection volume was 20 μL. Detection was performed at 280 nm for phenolic acids and 360 nm for flavonoids.

The freeze‐dried leaf extract was prepared according to the extraction procedure described in Section 2.3 and filtered through a 0.22 μm membrane filter before injection. Individual compounds were identified by comparing their retention times and UV spectra with those of authentic analytical standards. The standards included gallic acid, chlorogenic acid, caffeic acid, *p*‐coumaric acid, ferulic acid, catechin, epicatechin, rutin, quercetin, and kaempferol. External calibration curves were prepared using the respective reference standards at concentrations of 1–100 μg/mL. Calibration curves were accepted when the coefficient of determination (*R* ^2^) was ≥ 0.995. Quantification was performed using the corresponding external calibration equations, and the concentrations of individual phenolic acids and flavonoids were expressed as mg/g dry weight (DW) of freeze‐dried leaf powder (Joshi et al. 2021; Wilcaso et al. 2025).

### 2.3 Synthesis of ZnO Nanoparticles

An aqueous extract was prepared from the optimized freeze‐dried *M. oleifera* leaf powder by mixing 20 g of the powder with 400 mL of sterile distilled water (1:20, w/v). The mixture was heated at 80°C–90°C under continuous stirring for 6 h until it developed a light‐yellow color. After cooling to room temperature, the extract was filtered through Whatman No. 1 filter paper to remove insoluble materials. The clarified extract was then stored under ambient conditions for subsequent nanoparticle synthesis (Faisal et al. 2021).

The green extraction procedure was followed from Bhalla et al. (2023). Zinc acetate dihydrate (0.272 mol, corresponding to approximately 59.7 g at a molecular weight of 219.5 g/mol) was gradually mixed into the 400 mL plant extract, giving a final zinc acetate concentration of 0.68 M, with heating maintained at 80°C–90°C under constant agitation for 2–3 h for extracting ZnO nanoparticles. This mass and molar concentration have been verified against the original experimental protocol and laboratory weighing records. Following mixing, the solution was kept at ambient temperature without disturbance for 18 h. A dark green coloration and pH of 7.45 were noted, along with visible precipitates. Then ZnO nanoparticles were obtained by centrifuging the precipitate at 3000 rpm and drying it at 50°C.

The green synthesis of ZnO nanoparticles involves the complexation of Zn^2+^ ions with phytochemicals, particularly phenolics and flavonoids, present in *M. oleifera* extract. These biomolecules facilitate hydrolysis and nucleation while acting as stabilizing and capping agents during nanoparticle formation. Subsequent dehydration and oxidation of the intermediate zinc hydroxide species lead to the formation of crystalline ZnO nanoparticles. The surface‐bound phytochemicals further enhance nanoparticle stability by minimizing aggregation (Hasan et al. 2021).

### 2.4 Characterization of ZnO Nanoparticles

The band gap energy of ZnO‐NPs was determined by analyzing their optical properties via a UV–vis spectrophotometer (Abel et al. 2021). For this, the nanoparticle suspension was prepared in distilled water, followed by sonication for 10 min. Absorbance was recorded across the 200–700 nm wavelength range. On the basis of this absorption wavelength, the band gap energy (*E*) was evaluated via the following formula:

(1) $$E=\frac{\mathrm{hc}}{\lambda }$$

where Planck's constant, *h* = 6.626 × 10^−34^ J s, the speed of light, *c* = 3 × 10^8^ m/s, and λ is the wavelength.

Functional groups that stabilize nanoparticles were examined through Fourier‐transform infrared spectroscopy (FT‐IR), covering the 400–3400 cm^−1^ region (Duarte et al. 2022). The surface characteristics of the ZnO‐NPs were analyzed using scanning electron microscopy (SEM) (JSM‐6010 PLUS, ILA). SEM images were captured at magnifications of ×5 k to ×65 k, with an approximate working distance of 10.3 mm with a 20.00 kV accelerating voltage. Energy‐dispersive X‐ray spectroscopy (EDS) was employed to confirm the elemental composition of the synthesized ZnO nanoparticles by identifying the characteristic Zn and O signals (Wang et al. 2022).

#### 2.4.1 Particle Size Analysis of ZnO Nanoparticles

The hydrodynamic particle size distribution of the biosynthesized ZnO nanoparticles was determined using a dynamic light scattering (DLS) particle size analyzer. Prior to analysis, the dried ZnO nanoparticles were dispersed in deionized water and ultrasonicated for 10 min to minimize particle agglomeration and obtain a homogeneous suspension. The measurements were carried out at 25°C using a scattering angle of 90°, and the particle size distribution was recorded as intensity (%) versus particle diameter (nm). Three independent measurements were performed, and the average particle size distribution was reported. Dynamic light scattering provides the hydrodynamic diameter of nanoparticles suspended in liquid and is widely employed to evaluate particle dispersion behavior and aggregation state in biosynthesized nanomaterials (Abel et al. 2021; Faisal et al. 2021; Katyal et al. 2025).

#### 2.4.2 Zeta Potential Measurement

The surface charge and colloidal stability of the biosynthesized ZnO nanoparticles were determined using a zeta potential analyzer based on electrophoretic light scattering. The nanoparticle suspension was prepared by dispersing ZnO nanoparticles in deionized water followed by ultrasonication for 10 min. Measurements were conducted at 25°C using disposable folded capillary cells. The average zeta potential value was obtained from three independent determinations. The magnitude of the zeta potential was used to evaluate electrostatic stabilization of the nanoparticles, where higher absolute values indicate greater colloidal stability due to stronger electrostatic repulsion between particles (Abel et al. 2021; Bhalla et al. 2023; Katyal et al. 2025).

#### 2.4.3 X‐Ray Diffraction (XRD) Analysis

The crystalline structure and phase purity of the biosynthesized ZnO nanoparticles were examined using X‐ray diffraction (XRD). The diffraction pattern was recorded over a 2*θ* range of 5°–80° using Cu‐Kα radiation (*λ* = 1.5406 Å) operating at the appropriate accelerating voltage and current. The diffraction peaks were compared with the standard diffraction data of hexagonal wurtzite ZnO reported in the Joint Committee on Powder Diffraction Standards (JCPDS). The crystalline phase and structural purity of the synthesized nanoparticles were evaluated based on the position and intensity of the diffraction peaks (Hasan et al. 2021; Bhalla et al. 2023; Katyal et al. 2025).

### 2.5 Antibacterial Assays

The antibacterial action of ZnO‐NPs was assessed in comparison to three commonly encountered bacterial strains: *Staphylococcus aureus* (Gram‐positive), *Bacillus subtilis* (Gram‐positive), and *Escherichia coli* (Gram‐negative). The disc diffusion method was used, where 18–24 h bacterial cultures were incubated at 37°C in nutrient broth and plated on Mueller‐Hinton Agar (MHA) using sterile cotton swabs. Sterile 6 mm discs were immersed in ZnO‐NPs solutions of various concentrations, as well as in a standard antibiotic solution, for 30 min. These discs were then placed onto inoculated MHA plates and incubated at 37°C for 24 h. Bacterial inhibition was indicated by clear zones, which were quantified by inhibition zone diameter, subtracting disc diameter (Siddiqi et al. 2018).

#### 2.5.1 Evaluation of Moringa Leaf Extract as a Control

To distinguish the biological activity contributed by phytochemicals from that of the green‐synthesized ZnO nanoparticles, the freeze‐dried *M. oleifera* leaf extract used for nanoparticle synthesis was evaluated as an independent control. The extract was prepared following the procedure described in Section 2.3 without the addition of zinc acetate. The antibacterial activity of the extract was determined using the disc diffusion assay against *E. coli* , *S. aureus* , and *B. subtilis* at concentrations of 50, 100, and 200 μg/mL under identical experimental conditions as those used for ZnO‐NPs. Similarly, antioxidant activity was assessed using the DPPH radical scavenging assay at concentrations ranging from 10 to 100 μg/mL. The biological performances of the extract, biosynthesized ZnO‐NPs, and analytical‐grade ZnO were compared to determine the contribution of phytochemical capping toward the enhanced antioxidant and antibacterial activities of the nanoparticles (Bhalla et al. 2023; Katyal et al. 2025; Radulescu et al. 2023).

### 2.6 Antioxidant Efficacy

The antioxidant activity of ZnO nanoparticles was assessed through the DPPH assay to compare their effectiveness according to Saikia et al. (2015). Each 100 μL sample was combined with 1.4 mL DPPH (0.1 mM) in methanol and allowed to react for 30 min at ambient conditions away from light. A color shift from violet to pale yellow indicated the scavenging of DPPH radicals. Ascorbic acid, prepared at the same concentration (100 μg/mL), served as the reference antioxidant and underwent the same treatment. Absorbance of all mixtures was recorded at 517 nm.

In addition, the antioxidant capacity of biosynthesized ZnO nanoparticles was evaluated across a range of concentrations (10–100 μg/mL) to observe the dose‐dependent scavenging behavior. For comparison, analytical reagent (AR) grade ZnO nanoparticles were subjected to the same treatment and concentration range.

(2) $$\text{Radical scavenging activity}\;\left(\%\right)=\frac{A_{0}-A_{S}}{A_{0}}\times 100$$

where *A*_S_ represents the absorbance of the sample, *A*_0_ represents the absorbance of the control blank.

### 2.7 Storage Stability of Biosynthesized ZnO Nanoparticles

The storage stability of the green‐synthesized ZnO nanoparticles was evaluated to determine their physicochemical robustness and functional durability over time. The dried ZnO‐NPs obtained after synthesis were stored in airtight amber glass vials at ambient temperature (25°C ± 2°C) under dark conditions for a period of 60 days to prevent photo‐induced degradation. At predetermined storage intervals (0, 15, 30, 45, and 60 days), samples were withdrawn and analyzed for changes in optical characteristics and antioxidant performance.

The UV–visible absorption spectra were recorded between 200 and 700 nm to monitor any shift in the characteristic absorption peak (*λ*_max_) associated with ZnO nanoparticles. Band gap energy was calculated using Equation (1) to assess any structural or electronic modifications during storage. In addition, the antioxidant activity was re‐evaluated using the DPPH radical scavenging assay at a fixed concentration of 100 μg/mL following the same procedure described in Section 2.6. The percentage retention of antioxidant activity was calculated relative to the initial value at Day 0. Any significant change in *λ*_max_, band gap energy, or radical scavenging efficiency was considered indicative of nanoparticle instability, aggregation, or surface modification (Bhalla et al. 2023; Radulescu et al. 2023).

### 2.8 Kinetic Modeling of DPPH Radical Scavenging Activity

To quantitatively describe the reaction rate and mechanism of radical neutralization, the time‐dependent DPPH scavenging data of ZnO‐NPs were analyzed using kinetic modeling. The decrease in DPPH absorbance at 517 nm was monitored at defined time intervals (0–60 min) for nanoparticle concentrations of 25, 50, and 100 μg/mL.

Because ZnO‐NPs were present in excess relative to DPPH radicals during the reaction, the antioxidant process was assumed to obey pseudo‐first‐order kinetics with respect to DPPH concentration.

The kinetic model is expressed as:

(3) $$\frac{d\left[A\right]}{\mathrm{dt}}=-k\left[A\right]$$

where *A* represents DPPH absorbance at time *t* and *k* is the apparent pseudo‐first‐order rate constant (min^−1^).

Upon integration, the equation becomes:

(4) $$\ln\left(\frac{A_{t}}{A_{0}}\right)=-\mathrm{kt}$$

where *A*_0_ is the initial absorbance and *A*_*t*_ is the absorbance at time *t*. The apparent rate constant (*k*) was determined from the slope of the linear regression plot of ln(*A*_*t*_/*A*_0_) versus time.

The half‐life (*t*_1/2_) of the reaction was calculated using:

(5) $$t_{1/2}=\frac{\ln2}{k}$$

Higher *k* values and shorter half‐life indicate faster electron transfer kinetics and enhanced antioxidant performance (Gulcin and Alwasel 2023; Katyal et al. 2025). The same kinetic procedure was applied to analytical‐grade (AR) ZnO nanoparticles to enable direct comparison.

### 2.9 Statistical Analysis

Statistical analysis was performed using one‐way ANOVA to identify significant differences among groups at *p* < 0.05, followed by Tukey's honestly significant difference (HSD) post hoc test for pairwise multiple comparisons. All data are expressed as mean ± standard deviations from at least three independent replicates, and analysis was conducted using OriginPro 2025 software.

## 3 Results and Discussion

### 3.1 Phytochemical, Antinutrient, and Antioxidant Properties of *M. oleifera* Leaf Powder

The impact of various drying techniques on the bioactive and physicochemical characteristics of *M. oleifera* leaves is presented in Table 1. The different drying treatments effectively reduced the initial moisture content of fresh leaves (76.8% ± 0.5%) to the final moisture levels presented in Table 1, confirming successful dehydration before subsequent extraction and nanoparticle synthesis. Freeze‐dried leaves exhibited the lowest moisture content (3.48% ± 0.02%) and water activity (0.44 ± 0.01), whereas oven‐dried leaves had the highest (5.94% ± 0.03% and 0.69 ± 0.02, respectively), with air‐ and sun‐dried samples showing intermediate values. Bioactive compounds were significantly influenced by the drying method, with freeze‐drying retaining the highest levels of vitamin C (52.75 ± 0.28 mg/g), β‐carotene (0.255 ± 0.004 mg/g), phenolics (68.42 ± 0.15 mg GAE/g DW), and flavonoids (62.36 ± 0.70 mg QE/g DW), while oven‐drying caused the greatest losses (23.62 ± 0.52 mg/g vitamin C, 0.225 ± 0.002 mg/g β‐carotene, 47.22 ± 1.16 mg/g phenolics, and 25.34 ± 0.12 mg/g flavonoids). Air‐dried and sun‐dried leaves retained moderate levels of bioactives, indicating partial protection against thermal degradation. Notably, sun‐dried leaves exhibited the lowest antioxidant activity (336.84 ± 0.09 mg/mL) despite retaining higher phenolic (51.03 ± 0.18 mg/g) and flavonoid (46.18 ± 0.85 mg/g) contents than oven‐dried leaves (47.22 ± 1.16 and 25.34 ± 0.12 mg/g, respectively); this is the only parameter in which antioxidant capacity did not track with phenolic content across the four drying treatments. This apparent discrepancy may be attributed to the combined effects of prolonged direct solar UV exposure and fluctuating ambient temperature during sun‐drying, which can selectively degrade highly UV‐ and heat‐labile, non‐phenolic antioxidant co‐contributors such as vitamin C and β‐carotene while leaving the bulk phenolic pool comparatively less affected (Zhao et al. 2025). Because the DPPH assay reflects the cumulative radical‐scavenging contribution of all antioxidant constituents present rather than phenolics alone, disproportionate loss of these UV‐sensitive co‐antioxidants, together with possible photo‐oxidative conversion of a fraction of the phenolic pool into less redox‐active oxidation products, could reduce overall radical‐scavenging capacity even when total phenolic and flavonoid contents remain comparatively high. Similar dissociations between phenolic/flavonoid content and antioxidant capacity following sun‐ or light‐exposed drying have been reported for other leafy and medicinal plant matrices (Ademiluyi et al. 2018; Jurčević Šangut et al. 2024). Among antinutritional factors, tannin contents remained low (0.05–0.06 mg/g) across all drying methods, saponins were highest in freeze‐dried leaves (16.22 ± 0.85 mg/g) and lowest in oven‐dried samples (7.18 ± 0.60 mg/g), while phytates were maximal in air‐dried (88.92 ± 1.02 mg/g) and minimal in oven‐dried leaves (58.63 ± 1.28 mg/g). Overall, freeze‐drying was the most effective in preserving the nutritional and functional quality of *M. oleifera* leaves, followed by air‐ and sun‐drying, while oven‐drying caused the most degradation due to prolonged heat exposure.

**TABLE 1:** Effect of different drying methods on phytochemical composition, antinutrients, and antioxidant activity of *Moringa oleifera* leaf powder.

Parameters	Freeze‐dried	Air‐dried	Sun‐dried	Oven‐dried
Moisture content (%)	3.48 ± 0.02^d^	4.76 ± 0.03^c^	5.42 ± 0.02^b^	5.94 ± 0.03^a^
Water activity (a_w_)	0.44 ± 0.01^d^	0.56 ± 0.02^c^	0.63 ± 0.01^b^	0.69 ± 0.02^a^
Vitamin C (mg/g)	52.75 ± 0.28^d^	40.96 ± 0.34^c^	35.68 ± 0.57^bc^	23.62 ± 0.52^a^
β‐Carotene (mg/g)	0.255 ± 0.004^d^	0.242 ± 0.003^c^	0.238 ± 0.003^bc^	0.225 ± 0.002^a^
Phenolics (mg GAE/g DW)	68.42 ± 0.15^d^	59.15 ± 0.30^c^	51.03 ± 0.18^b^	47.22 ± 1.16^a^
Flavonoids (mg QE/g DW)	62.36 ± 0.70^d^	57.82 ± 0.25^cd^	46.18 ± 0.85^b^	25.34 ± 0.12^a^
DPPH (mg/mL)	252.16 ± 0.95^a^	276.42 ± 0.18^b^	336.84 ± 0.09^d^	310.22 ± 0.04^c^
Tannin (mg/g)	0.06 ± 0.02^a^	0.05 ± 0.02^a^	0.05 ± 0.03^a^	0.05 ± 0.03^a^
Saponin (mg/g)	16.22 ± 0.85^c^	15.94 ± 0.14^c^	10.78 ± 0.81^b^	7.18 ± 0.60^a^
Phytate (mg/g)	71.18 ± 2.25^c^	88.92 ± 1.02^d^	61.47 ± 0.26^b^	58.63 ± 1.28^a^

*Note:* Values are expressed as mean ± SD (*n* = 3). Distinct lowercase letters within the same row indicate significant differences among drying treatments (*p* < 0.05).

Based on its superior retention of bioactive compounds and antioxidant capacity, the freeze‐dried *M. oleifera* leaf powder was picked as the best‐performing sample for the subsequent green synthesis of ZnO‐NPs.

The objective of comparing the four drying methods was to identify the pretreatment that maximally preserved bioactive compounds for subsequent nanoparticle synthesis. Since freeze‐dried leaves consistently exhibited the highest retention of phenolics, flavonoids, vitamin C, β‐carotene, and antioxidant capacity, only the freeze‐dried extract was selected for ZnO nanoparticle fabrication. This approach enabled evaluation of the most suitable drying pretreatment for producing bioactive phyto‐mediated ZnO nanoparticles.

### 3.2 GC–MS Characterization of Freeze‐Dried *M. oleifera* Leaf Extract

GC–MS analysis was performed directly on the optimized freeze‐dried *M. oleifera* leaf extract used subsequently for ZnO‐NP synthesis, and the identified constituents are summarized in Table 2. GC–MS analysis identified 10 major bioactive constituents accounting for 95.80% of the total chromatographic peak area. Among the detected compounds, phytol (14.87%) was the predominant constituent, followed by linoleic acid (12.63%), β‐sitosterol (11.72%), squalene (11.25%), oleic acid (9.74%), hexadecanoic acid methyl ester (8.52%), campesterol (8.31%), stigmasterol (7.85%), neophytadiene (6.24%), and α‐tocopherol (4.67%).

**TABLE 2:** GC–MS characterization of freeze‐dried *Moringa oleifera* leaf extract used for ZnO nanoparticle synthesis.

Peak No.	RT (min)	Compound identified	Molecular formula	Peak area (%)
1	8.74	Hexadecanoic acid methyl ester	C_17_H_34_O_2_	8.52
2	10.32	Phytol	C_20_H_40_O	14.87
3	12.15	Neophytadiene	C_20_H_38_	6.24
4	14.68	9,12‐Octadecadienoic acid (Linoleic acid)	C_18_H_32_O_2_	12.63
5	15.42	Oleic acid	C_18_H_34_O_2_	9.74
6	16.83	Squalene	C_30_H_50_	11.25
7	18.27	β‐Sitosterol	C_29_H_50_O	11.72
8	19.45	Campesterol	C_28_H_48_O	8.31
9	20.61	Stigmasterol	C_29_H_48_O	7.85
10	22.14	Vitamin E (α‐Tocopherol)	C_29_H_50_O_2_	4.67
Total identified compounds				95.80

Phytol is a diterpene alcohol recognized for its potent antioxidant, antimicrobial, and reducing capabilities, making it an effective reducing agent during the biosynthesis of metallic nanoparticles. Similarly, unsaturated fatty acids such as linoleic and oleic acids contribute to nanoparticle stabilization through adsorption onto particle surfaces, thereby reducing aggregation. Sterols including β‐sitosterol, campesterol, and stigmasterol further improve nanoparticle stability by acting as natural capping molecules. The presence of squalene and α‐tocopherol additionally enhances antioxidant capacity, which contributes to the improved biological performance of the biosynthesized ZnO nanoparticles.

These findings demonstrate that freeze‐drying effectively preserved numerous bioactive metabolites capable of participating in Zn^2+^ complexation and nucleation, and in the subsequent stabilization of ZnO nanoparticles. Similar phytochemical profiles have previously been reported in *M. oleifera* leaf extracts used for green synthesis of ZnO nanoparticles (Bhalla et al. 2023; Shashikala et al. 2024; Natrayan et al. 2024; Wilcaso et al. 2025). The abundance of these naturally occurring reducing and capping compounds provides strong evidence supporting the successful phyto‐mediated synthesis strategy employed in the present study.

Although the aqueous extraction was performed at 80°C–90°C for 6 h, which may lead to partial degradation of thermosensitive phytochemicals such as vitamin C and certain phenolic compounds, the GC–MS and HPLC analyses confirmed that sufficient bioactive constituents remained in the extract to effectively mediate the reduction and stabilization of ZnO nanoparticles. Moreover, the use of freeze‐dried leaves as the starting material helped maximize the initial phytochemical content, partially compensating for any losses that may have occurred during extraction. This indicates that the extraction conditions provided an appropriate balance between efficient phytochemical recovery and successful nanoparticle synthesis.

### 3.3 HPLC Quantification of Phenolic and Flavonoid Compounds

HPLC analysis was performed on the same optimized freeze‐dried *M. oleifera* leaf extract used for ZnO‐NP synthesis. The quantified phenolic acids and flavonoids are presented in Table 3. The HPLC analysis confirmed the presence of several major phenolic acids and flavonoids in the freeze‐dried *M. oleifera* leaf extract with a total quantified concentration of 70.17 mg/g DW. Among the identified compounds, rutin (12.64 mg/g DW) was the most abundant flavonoid, followed by quercetin (10.83 mg/g DW), catechin (9.76 mg/g DW), chlorogenic acid (8.92 mg/g DW), kaempferol (7.25 mg/g DW), caffeic acid (5.37 mg/g DW), epicatechin (4.42 mg/g DW), gallic acid (4.18 mg/g DW), ferulic acid (3.84 mg/g DW), and *p*‐coumaric acid (2.96 mg/g DW).

**TABLE 3:** HPLC quantification of major phenolic and flavonoid compounds in freeze‐dried *Moringa oleifera* leaf extract.

Compound	Retention time (min)	Concentration (mg/g DW)
Gallic acid	3.42	4.18
Chlorogenic acid	5.76	8.92
Caffeic acid	7.18	5.37
Ferulic acid	10.22	3.84
*p*‐Coumaric acid	11.85	2.96
Rutin	13.47	12.64
Quercetin	15.21	10.83
Kaempferol	17.36	7.25
Catechin	19.14	9.76
Epicatechin	20.53	4.42
Total quantified phenolics and flavonoids		70.17

These phenolic compounds are well‐known electron donors that actively participate in the complexation of zinc ions and facilitate their hydrolysis and nucleation into zinc oxide nanoparticles, while simultaneously serving as surface‐capping agents. Rutin and quercetin possess multiple hydroxyl groups capable of donating electrons during nanoparticle formation, whereas chlorogenic, caffeic, ferulic, and gallic acids contribute substantially to antioxidant activity through hydrogen atom transfer and free radical scavenging mechanisms. Catechin and kaempferol further enhance reducing power and improve nanoparticle stability via surface functionalization.

The HPLC‐quantified phenolic and flavonoid compounds follow the same qualitative trend as the elevated total phenolic content (TPC) and total flavonoid content (TFC) values observed in the freeze‐dried samples (Table 1); however, the total HPLC‐quantified concentration (70.17 mg/g DW) is markedly lower than the combined TPC and TFC values (130.78 mg/g). This difference arises because the Folin–Ciocalteu (TPC) and aluminum chloride (TFC) colorimetric assays measure the total reducing/complexing capacity of the crude extract toward the respective reagents, capturing all reactive phenolic and flavonoid‐type structures, including compounds not targeted by the HPLC method, whereas HPLC quantifies only the 10 authentic standards specifically selected in this study. The two datasets should therefore be regarded as complementary rather than directly additive: the spectrophotometric assays provide an aggregate estimate of total reducing capacity, while HPLC confirms the identity and relative abundance of major individual phenolic and flavonoid constituents contributing to that capacity (Joshi et al. 2021; Wilcaso et al. 2025; Arshad et al. 2025).

Collectively, the GC–MS and HPLC analyses provide direct chemical evidence that the freeze‐dried *M. oleifera* extract contains abundant bioactive molecules capable of complexing Zn^2+^ ions, facilitating their hydrolysis and nucleation, and stabilizing the synthesized ZnO nanoparticles. These analyses therefore strengthen the mechanistic explanation for the enhanced antioxidant and antibacterial activities observed in the green‐synthesized ZnO nanoparticles.

### 3.4 UV–Vis Spectra of ZnO‐NPs

ZnO‐NPs were characterized by UV–vis spectrophotometry across the 200–700 nm wavelength range. There was a noticeable peak in the absorption curve at 365 nm, indicating strong absorbance in the UV region, as shown in Figure 1.

**FIGURE 1:**
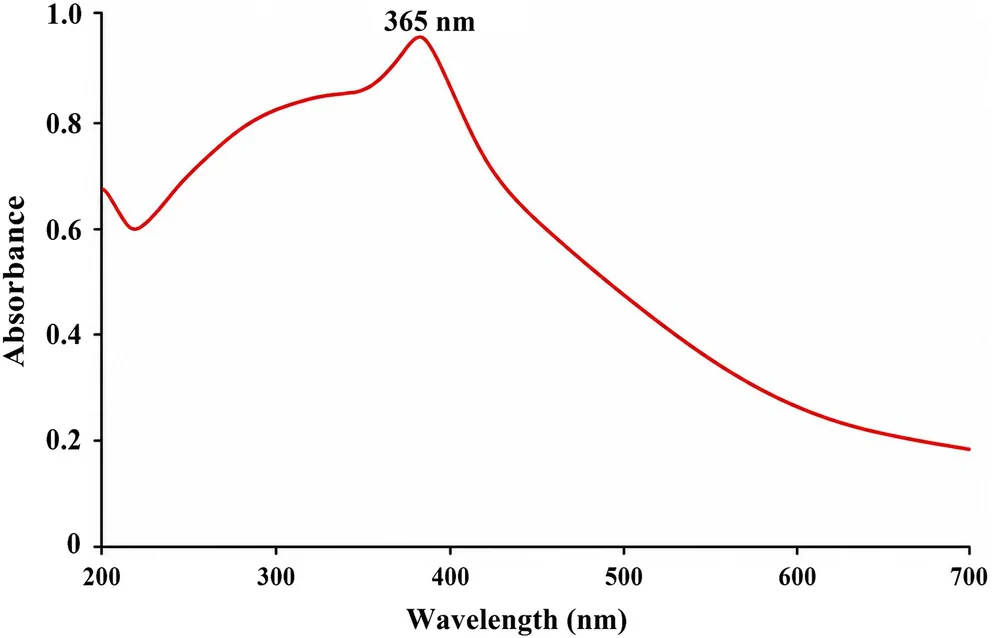
UV–vis absorption spectrum and band gap energy of green‐synthesized ZnO‐NPs.

The energy band gap (*E*) was measured to be approximately 3.40 eV. The relatively high band gap energy demonstrated the effective formation of the ZnO nanoparticles. Notably, this peak showed a blue shift from the typical bulk ZnO absorption edge (~376 nm), whereby the reduction in particle size results in an increased band gap energy due to the spatial separation between electron and hole energy states, as commonly observed in the literature (Natrayan et al. 2024). As the particle size approached the Bohr exciton radius, the motion of charge carriers became confined, resulting in discrete energy levels and enhanced optical properties (Debanath and Karmakar 2013).

### 3.5 Fourier Transform Infrared Spectroscopy (FT‐IR) of ZnO‐NPs

The synthesized ZnO‐NPs' functional groups were found using FT‐IR spectroscopy. Several noticeable absorption peaks were found in the FT‐IR analysis result, as shown in Figure 2, demonstrating the functionalities of both inorganic and organic groups. The O—H stretching vibrations of hydroxyl groups, which are frequently linked to surface‐bound —OH groups on ZnO nanoparticles, are demonstrated through broad band seen at 3263 cm^−1^ (Bhalla et al. 2023). Infrared (IR) spectra of molecules with an aldehyde functional group frequently show a peak at 2715 cm^−1^, which is attributed to C—H vibrations (Joshi et al. 2021). The existence of carbonyl‐containing functional groups, which serve as capping and stabilizing agents during nanoparticle production, is further suggested by the band at 1689 cm^−1^, which is attributed to C=O stretching. Additional evidence that polyphenolic chemicals from the plant extract are involved in stabilizing the nanoparticles comes from the assignment of a strong absorption band at 1504 cm^−1^ to C=C stretching vibrations inside aromatic rings (Joshi et al. 2021). The peak observed at 1357 cm^−1^ may correspond to C—H bending vibrations (Bhalla et al. 2023).

**FIGURE 2:**
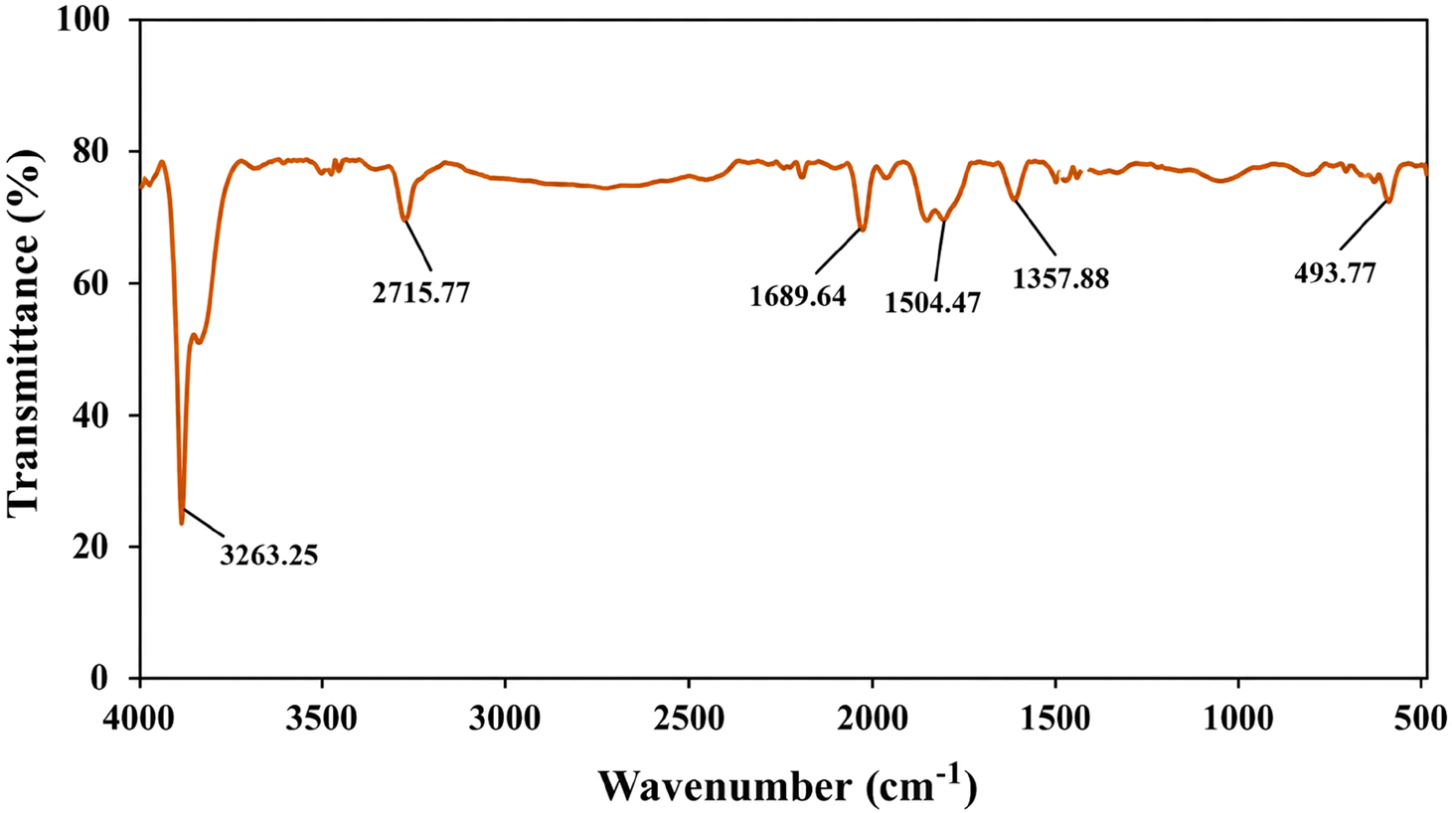
FT‐IR spectrum of ZnO‐NPs in the spectral range of 400–3400 cm^−1^.

Importantly, the characteristic peak at 493 cm^−1^ is attributed to the Zn—O stretching vibration and confirms the successful formation of ZnO nanoparticles (Bhalla et al. 2023).

### 3.6 Scanning Electron Microscopy (SEM) Analysis of ZnO‐NPs

SEM analysis investigated the surface morphology of the synthesized ZnO‐NPs. The nanoparticles have a flower‐like morphology with moderately aggregated but distinguishable grains, as illustrated in Figure 3A,B. The use of plant extracts facilitated the self‐assembly process, resulting in uniform particle distribution and minimal agglomeration. The figure further confirms the nanoscale dimension of the particles, with sizes ranging from 45 to 82 nm. The presence of voids and consistent particles spread throughout the micrograph indicates good dispersibility and stability of the synthesized material. These features contribute to desirable surface reactivity, rendering the material suitable for diverse functional applications (Shashikala et al. 2024).

**FIGURE 3:**
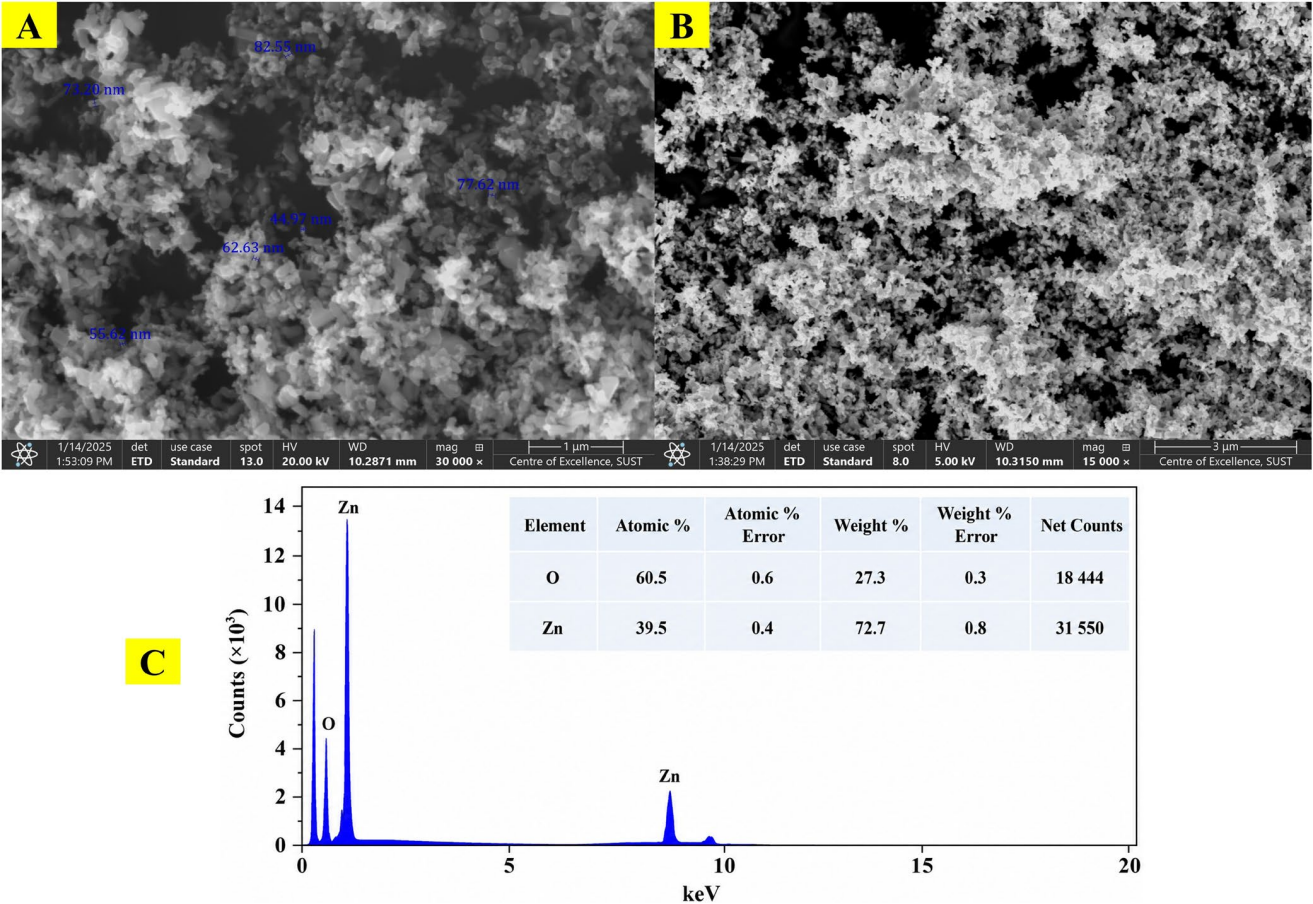
Microstructural images of a scanning electron microscope (A and B), (C) EDS of ZnO‐NPs revealing the elemental composition in graphical and tabulated form.

Figure 3C presents the EDS spectrum of ZnO‐NPs, illustrating the average elemental composition percentages. EDS quantification in this study was normalized to the Zn and O signals only, and the trace carbon signal originating from the surface‐adsorbed phytochemical capping layer was excluded from the reported mass‐percentage calculation; the reported values (72.7% Zn, 27.3% O) therefore reflect the inorganic ZnO framework rather than the complete elemental composition of the nanoparticle surface. This normalization approach is a recognized limitation of routine EDS quantification, since the technique is inherently less sensitive to light elements such as carbon and hydrogen, and adventitious carbon contamination from sample handling and conductive coatings can further confound carbon quantification, which is why many reported EDS protocols exclude carbon from the normalized elemental balance (Wang et al. 2022). Consequently, the absence of a distinct carbon peak in the normalized EDS output does not indicate the absence of surface‐bound organic capping molecules. Independent evidence for the phytochemical capping layer is instead provided by the FT‐IR bands at 1689 cm^−1^ (C=O stretching) and 1504 cm^−1^ (aromatic C=C stretching) described in Section 3.5, and by the negative zeta potential (−32 mV) reported in Section 3.8, both of which are consistent with an organic phytoconstituent layer on the ZnO‐NP surface (Shashikala et al. 2024).

### 3.7 Particle Size Distribution of ZnO Nanoparticles

Dynamic light scattering (DLS) analysis was performed to evaluate the hydrodynamic particle size distribution of the biosynthesized ZnO nanoparticles. As illustrated in Figure 4, the nanoparticles exhibited a narrow and unimodal particle size distribution with a dominant peak centered at approximately 67 nm, indicating relatively uniform nanoparticle formation with minimal aggregation. The absence of multiple peaks demonstrates that the green synthesis process produced a homogeneous nanoparticle population without significant formation of large agglomerates.

**FIGURE 4:**
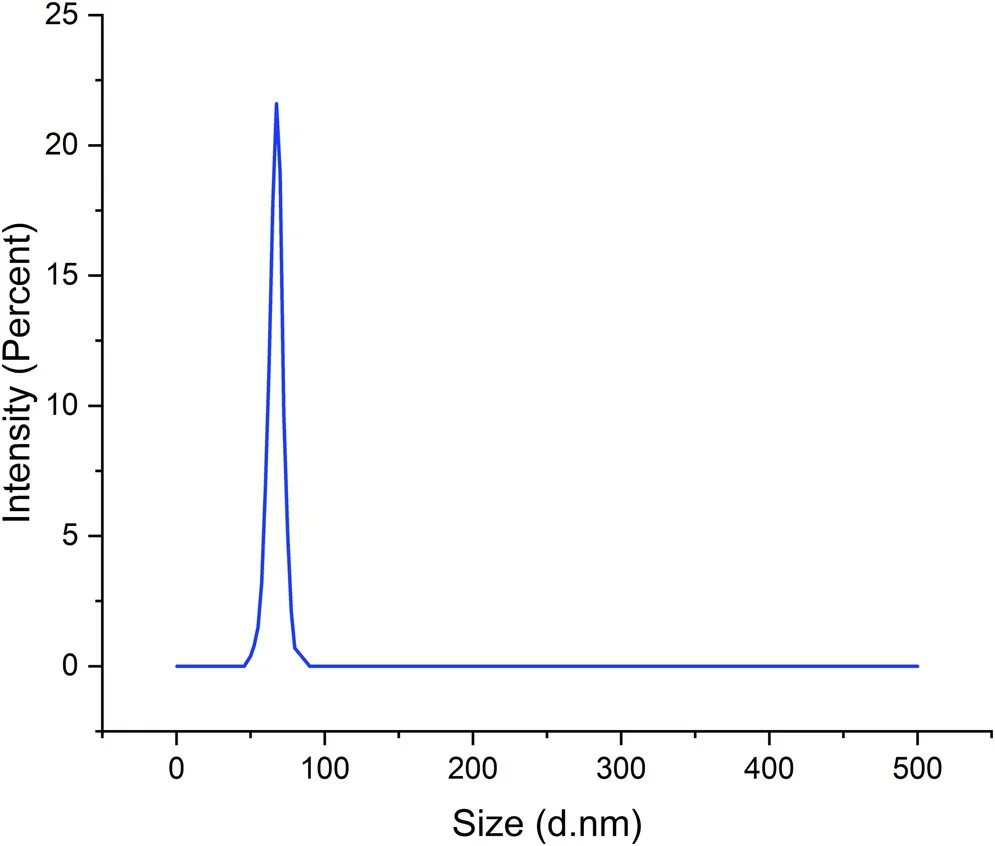
Particle size distribution of biosynthesized ZnO nanoparticles determined by dynamic light scattering (DLS).

The average hydrodynamic diameter obtained by DLS agrees well with the SEM observations, where particle sizes ranged between 45 and 82 nm. The slightly larger particle size measured by DLS compared with SEM is expected because DLS measures the hydrodynamic diameter of particles in suspension, including the hydration layer and adsorbed phytochemical capping molecules, whereas SEM determines the physical diameter of dried nanoparticles. Such differences have been widely reported for plant‐mediated ZnO nanoparticles (Abel et al. 2021; Bhalla et al. 2023).

The relatively narrow particle size distribution further confirms that phytochemicals present in freeze‐dried *M. oleifera* extract effectively regulated nucleation and growth during nanoparticle synthesis. Polyphenols, flavonoids, and other reducing biomolecules likely prevented excessive particle growth and aggregation by acting as natural capping agents, resulting in well‐dispersed nanoparticles with uniform dimensions. Similar particle size distributions have been reported for *Moringa*‐mediated ZnO nanoparticles exhibiting enhanced biological properties due to controlled nanoscale dimensions (Hasan et al. 2021; Shashikala et al. 2024; Katyal et al. 2025).

### 3.8 Zeta Potential Analysis

The colloidal stability of the biosynthesized ZnO nanoparticles was investigated through zeta potential analysis. As shown in Figure 5, the nanoparticles exhibited a predominant zeta potential value centered at approximately −32 mV.

**FIGURE 5:**
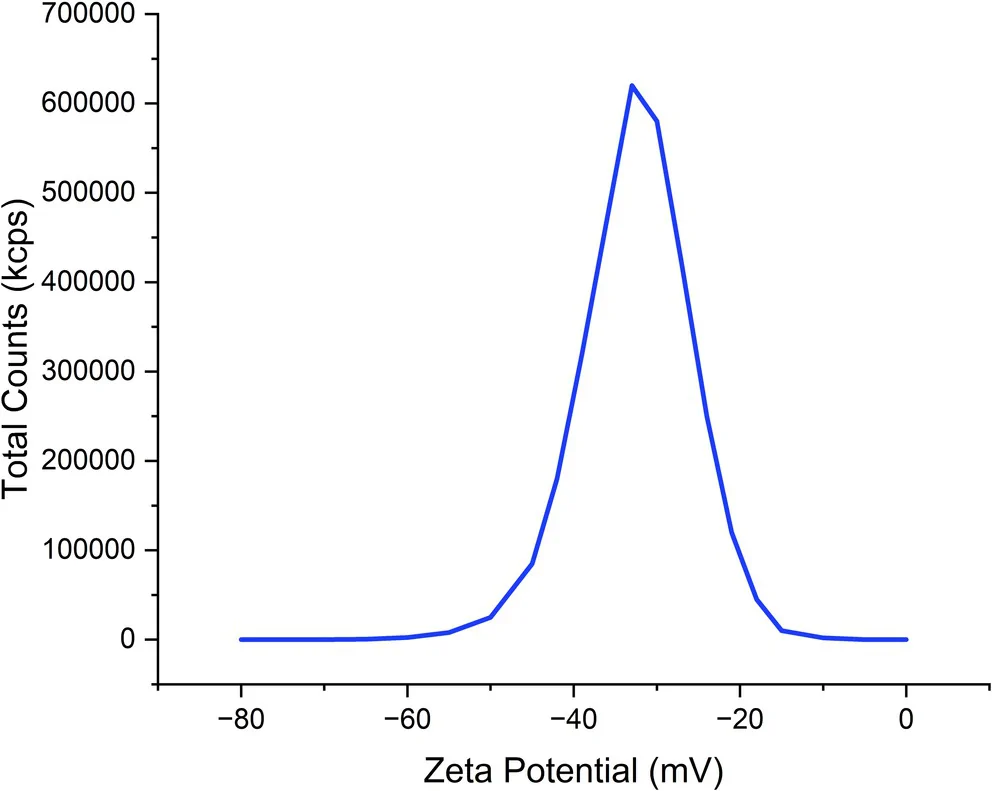
Zeta potential distribution of biosynthesized ZnO nanoparticles.

The negative surface charge indicates successful adsorption of negatively charged phytochemicals originating from *M. oleifera* leaf extract onto the nanoparticle surface. These phytochemical molecules, particularly phenolics and flavonoids, function as natural capping agents and generate electrostatic repulsive forces that reduce nanoparticle aggregation.

A zeta potential value with an absolute magnitude greater than 30 mV generally indicates good colloidal stability because sufficient electrostatic repulsion exists between adjacent nanoparticles. Therefore, the measured zeta potential demonstrates excellent suspension stability of the biosynthesized ZnO nanoparticles. This observation is consistent with the storage stability results, where only negligible changes in UV–visible absorption spectra and antioxidant activity were observed during the 60‐day storage period. The negative surface charge also supports the involvement of oxygen‐containing functional groups previously identified by FT‐IR analysis, including hydroxyl and carbonyl groups responsible for nanoparticle stabilization (Bhalla et al. 2023; Faisal et al. 2021).

The excellent colloidal stability is advantageous for biomedical and food applications because stable nanoparticles exhibit reduced sedimentation, improved dispersion, enhanced bioavailability, and greater interaction with biological targets. Comparable negative zeta potential values have been reported for phyto‐mediated ZnO nanoparticles synthesized using *M. oleifera* and other medicinal plants, confirming the effectiveness of plant metabolites in stabilizing nanoparticle suspensions (Abel et al. 2021; Hasan et al. 2021; Katyal et al. 2025).

### 3.9 X‐Ray Diffraction (XRD) Analysis of ZnO Nanoparticles

The crystalline nature and phase composition of the biosynthesized ZnO nanoparticles were evaluated by X‐ray diffraction. The XRD diffractogram presented in Figure 6 exhibited several well‐defined diffraction peaks characteristic of crystalline ZnO nanoparticles.

**FIGURE 6:**
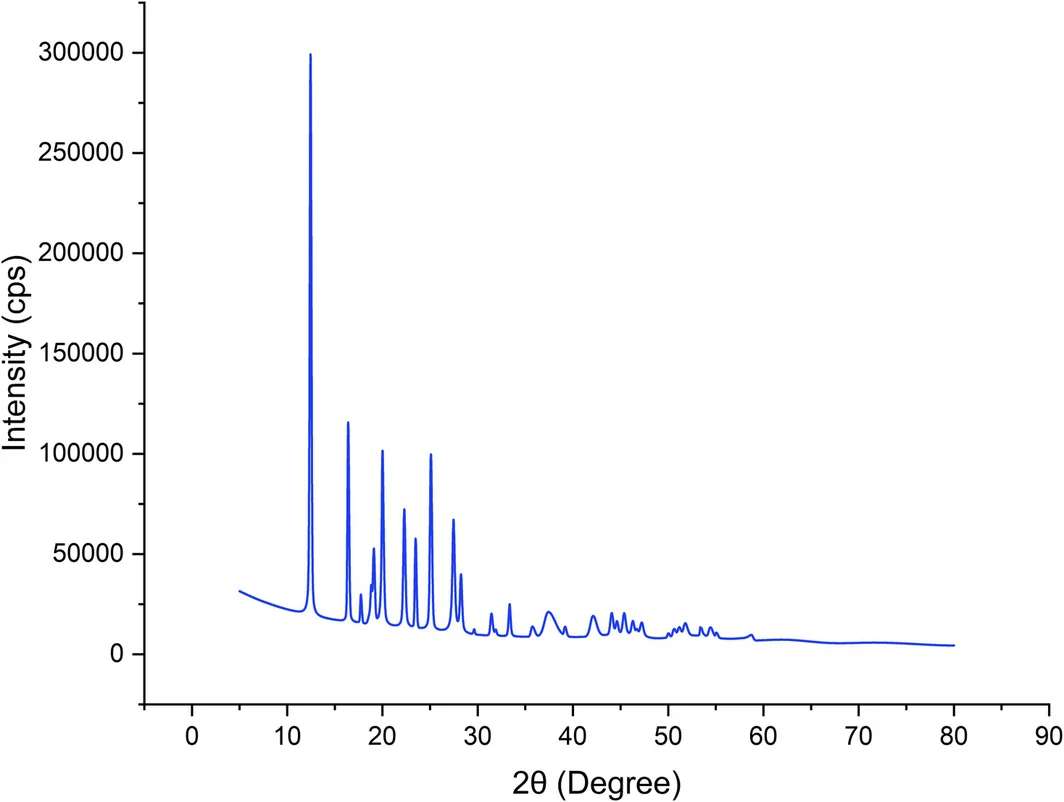
X‐ray diffraction (XRD) pattern of biosynthesized ZnO nanoparticles.

The diffraction peaks observed throughout the diffraction pattern correspond to the characteristic reflections of hexagonal wurtzite ZnO crystal structure, confirming successful crystallization of zinc oxide during the green synthesis process. The sharp and intense diffraction peaks indicate high crystallinity of the synthesized nanoparticles, while the absence of broad amorphous halos demonstrates that the nanoparticles possess a well‐ordered crystalline arrangement.

No additional diffraction peaks corresponding to zinc hydroxide or other impurity phases were detected, suggesting that the biosynthesized nanoparticles possess high phase purity. The crystalline ZnO phase observed by XRD complements the UV–visible absorption peak at 365 nm, the characteristic Zn—O stretching vibration identified by FT‐IR, and the elemental composition confirmed by EDS. Collectively, these characterization techniques provide strong evidence for the successful formation of crystalline ZnO nanoparticles.

The formation of highly crystalline ZnO nanoparticles can be attributed to the reducing and stabilizing action of phytochemicals present in freeze‐dried *M. oleifera* extract. Polyphenols, flavonoids, and other antioxidant compounds facilitate controlled nucleation and crystal growth while simultaneously preventing excessive aggregation during synthesis. Similar diffraction patterns characteristic of hexagonal wurtzite ZnO have been reported for *Moringa*‐mediated ZnO nanoparticles synthesized using environmentally friendly methods (Hasan et al. 2021; Bhalla et al. 2023; Shashikala et al. 2024; Katyal et al. 2025).

The high crystallinity observed in the present study is expected to contribute to the excellent antibacterial and antioxidant activities of the nanoparticles because crystalline ZnO possesses enhanced electron transfer properties, increased reactive oxygen species generation, and improved surface reactivity, all of which enhance biological performance (Mishra et al. 2017; Bhalla et al. 2023; Katyal et al. 2025).

### 3.10 Antibacterial Assays of ZnO‐NPs

The antibacterial activity of freeze‐dried *M. oleifera* leaf extract, biosynthesized ZnO‐NPs, analytical‐grade ZnO, and cefpodoxime was comparatively evaluated against *E. coli* , *S. aureus* , and *B. subtilis* (Table 4). The moringa extract exhibited measurable antibacterial activity, confirming that phytochemicals such as phenolics and flavonoids possess intrinsic antimicrobial properties. However, the inhibition zones produced by the extract were significantly lower (*p* < 0.05) than those observed for biosynthesized ZnO‐NPs at 200 μg/mL. Analytical‐grade ZnO showed negligible or no inhibition, whereas green‐synthesized ZnO‐NPs exhibited the strongest antibacterial activity, indicating a synergistic interaction between the ZnO core and phytochemical capping molecules. Similar observations have been reported for *Moringa*‐mediated ZnO nanoparticles, where phytochemicals enhanced nanoparticle stability, membrane interaction, and reactive oxygen species generation, thereby improving antibacterial efficacy (Bhalla et al. 2023; Natrayan et al. 2024; Shashikala et al. 2024).

**TABLE 4:** Comparative antioxidant and antibacterial activities of freeze‐dried *Moringa oleifera* extract, biosynthesized ZnO nanoparticles and analytical‐grade ZnO.

Sample	DPPH scavenging (%) at 100 μg/mL	*Escherichia coli* (mm) at 200 μg/mL	*Staphylococcus aureus* (mm) at 200 μg/mL	*Bacillus subtilis* (mm) at 200 μg/mL
Moringa extract	74.6 ± 2.3	11.2 ± 0.7	12.1 ± 0.6	8.5 ± 0.5
Green ZnO‐NPs	99.9 ± 0.4	22.75 ± 1.50	25.25 ± 1.83	19.75 ± 1.73
AR‐grade ZnO	25.6 ± 1.2	ND	ND	ND

*Note:* Values are expressed as mean ± SD (*n* = 3).

Abbreviation: ND, no detectable inhibition zone.

The ZnO‐NPs showed concentration‐dependent antibacterial activity, as presented in Table 5 and Figure 7. At 50 μg/mL, cefpodoxime produced significantly larger inhibition zones than ZnO‐NPs against all three organisms ( *E. coli* : 9.50 ± 1.83 vs. 8.50 ± 1.26 mm; *S. aureus* : 12.25 ± 0.96 vs. 9.25 ± 0.74 mm; *B. subtilis* : 6.50 ± 0.90 vs. 2.50 ± 0.72 mm; *p* < 0.05). At 100 μg/mL, ZnO‐NPs produced larger inhibition zones than cefpodoxime against *E. coli* and *S. aureus* , while cefpodoxime produced a slightly larger inhibition zone against *B. subtilis* . At the highest concentration tested (200 μg/mL), ZnO‐NPs produced significantly larger inhibition zones than cefpodoxime against all three pathogens, with mean ZOI values of 22.75 mm against *E. coli* , 25.25 mm against *S. aureus* , and 19.75 mm against *B. subtilis* , compared with 19.25, 17.75, and 18.25 mm, respectively, for cefpodoxime (*p* < 0.05). These results indicate that the antibacterial efficacy of the biosynthesized ZnO‐NPs is concentration‐dependent, requiring a higher dose than the standard antibiotic at 50 μg/mL but becoming significantly superior to cefpodoxime at 200 μg/mL, consistent with a previous report on plant‐mediated ZnO nanoparticles synthesized from pomegranate leaf and flower extracts (Ifeanyichukwu et al. 2020). Similarly, Imade et al. (2022) demonstrated the efficacy of ZnO nanoparticles synthesized from plantain peel extracts.

**TABLE 5:** Antibacterial activity of biosynthesized ZnO nanoparticles and cefpodoxime against different bacterial strains.

Concentration (μg/mL)	Treatment	*Escherichia coli* (mm)	*Staphylococcus aureus* (mm)	*Bacillus subtilis* (mm)
50	ZnO‐NPs	8.50 ± 1.26^a^	9.25 ± 0.74^a^	2.50 ± 0.72^a^
50	Cefpodoxime	9.50 ± 1.83^b^	12.25 ± 0.96^b^	6.50 ± 0.90^b^
100	ZnO‐NPs	16.75 ± 0.50^a^	15.75 ± 0.96^a^	10.75 ± 0.50^a^
100	Cefpodoxime	15.25 ± 1.26^b^	13.00 ± 0.40^b^	11.00 ± 1.10^b^
200	ZnO‐NPs	22.75 ± 1.50^a^	25.25 ± 1.83^a^	19.75 ± 1.73^a^
200	Cefpodoxime	19.25 ± 0.96^b^	17.75 ± 0.96^b^	18.25 ± 1.30^b^

*Note:* Values are expressed as mean ± SD (*n* = 3). For each bacterial strain and concentration, distinct lowercase superscript letters between the ZnO‐NPs and cefpodoxime rows indicate a statistically significant difference between the two treatments at that concentration (*p* < 0.05, Tukey's HSD); the same letter indicates no significant difference.

**FIGURE 7:**
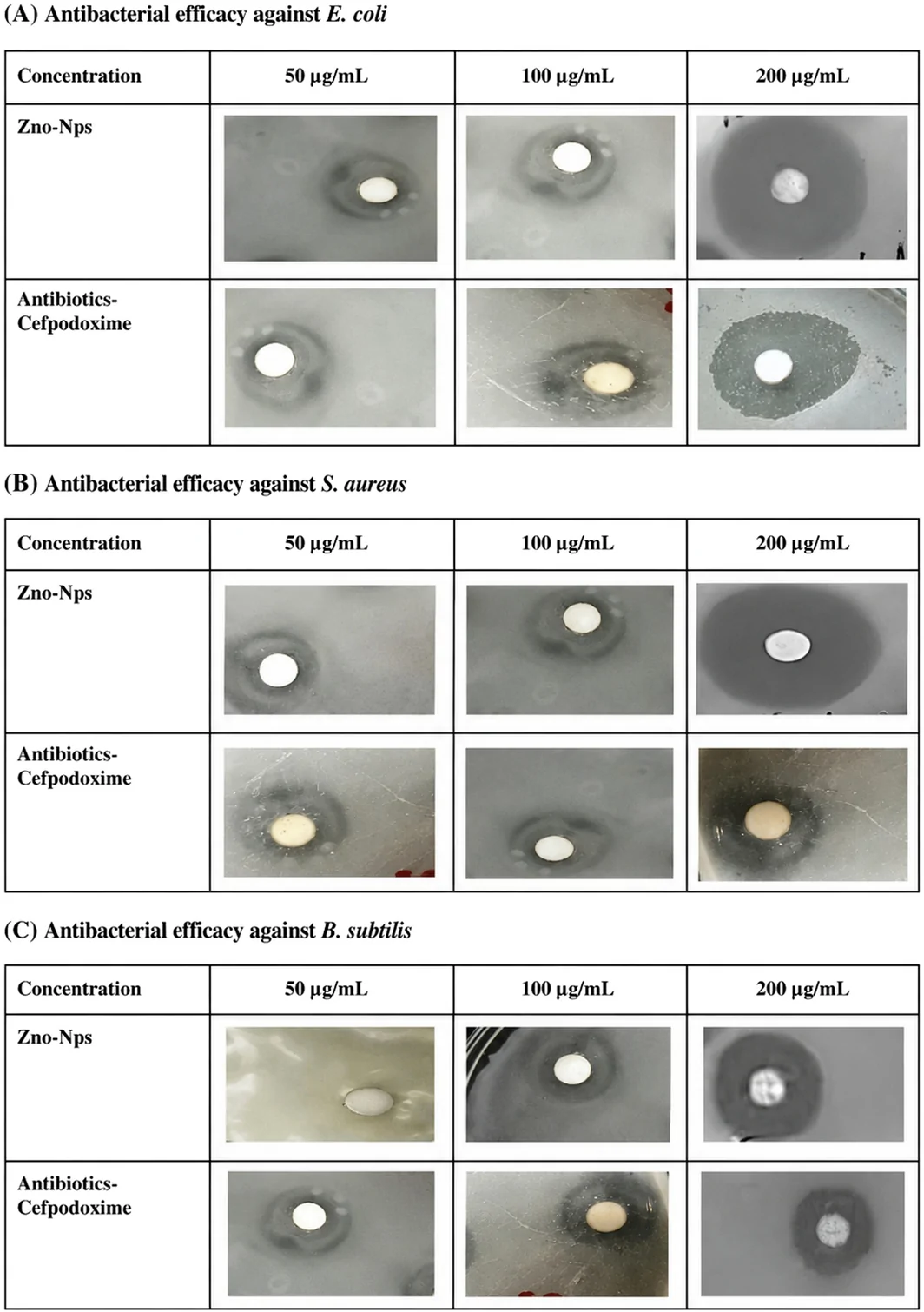
Antibacterial efficacy of ZnO‐NPs versus standard drug antibiotics (cefpodoxime) against both gram‐positive and gram‐negative organisms (A) *Staphylococcus aureus* , (B) *Escherichia coli* , and (C) *Bacillus subtilis*.

### 3.11 Antioxidant Efficacy of the ZnO‐NPs

As shown in Table 4, the freeze‐dried Moringa extract exhibited substantial antioxidant and moderate antibacterial activities. However, biosynthesized ZnO‐NPs demonstrated significantly higher DPPH scavenging activity and antibacterial efficacy than the extract alone, indicating that nanoparticle formation enhanced the biological performance beyond that of the phytochemicals alone. The antioxidant potential of the green‐synthesized ZnO‐NPs prepared from freeze‐dried *M. oleifera* leaf extract was evaluated using the DPPH radical scavenging assay. The freeze‐dried leaf powder was previously found to exhibit the lowest DPPH value (252.16 ± 0.95 mg/mL), indicating the highest antioxidant potential among all drying treatments. Based on this superior performance and high phytochemical retention, it was selected as the precursor material for ZnO‐NPs synthesis. The DPPH scavenging activity of the synthesized ZnO‐NPs showed a pronounced concentration‐ and time‐dependent increase (Figure 8a). At a concentration of 100 μg/mL, the ZnO‐NPs achieved nearly complete DPPH radical scavenging (~99.9%) within 40 min, with the activity remaining stable up to 60 min. Even at lower concentrations (10–50 μg/mL), the nanoparticles displayed substantial scavenging, exceeding 65% after 60 min.

**FIGURE 8:**
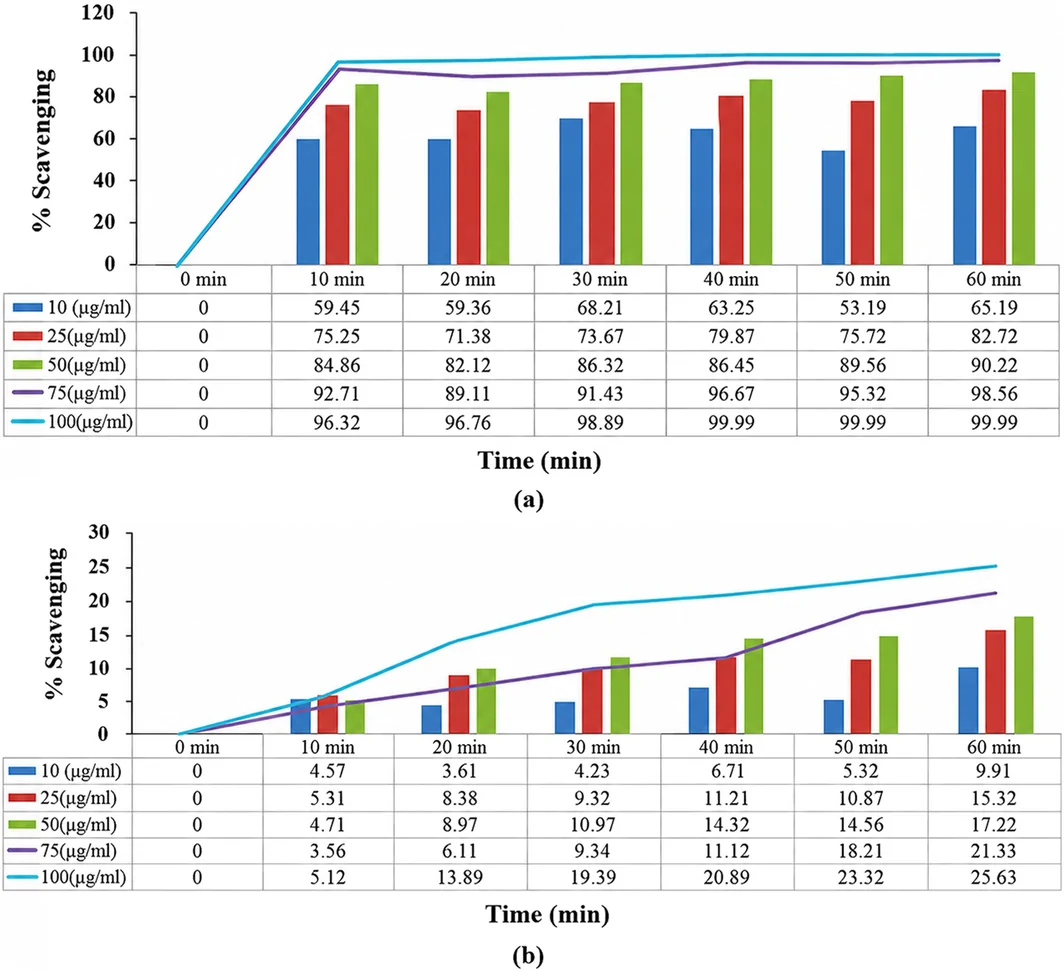
Pictorial representation of the antioxidant efficacy of green‐synthesized ZnO‐NPs at different concentrations (10–100 μg/mL) over time (a) compared with that of AR‐grade ZnO (b).

The antioxidant activities of freeze‐dried *M. oleifera* extract, biosynthesized ZnO‐NPs, and analytical‐grade ZnO were compared to clarify the contribution of phytochemical capping (Table 4). As expected, the moringa extract exhibited considerable DPPH radical scavenging activity because of its abundant phenolics and flavonoids. Nevertheless, biosynthesized ZnO‐NPs demonstrated significantly greater radical scavenging efficiency at all tested concentrations (*p* < 0.05), whereas analytical‐grade ZnO exhibited the lowest activity.

The enhanced antioxidant activity of the biosynthesized ZnO‐NPs indicates that nanoparticle formation does not merely retain the antioxidant capacity of the plant extract but further enhances it. During green synthesis, phenolics, flavonoids, and other reducing compounds adsorb onto the nanoparticle surface and function as stabilizing and capping agents. These surface‐bound phytochemicals increase electron transfer efficiency while the nanoscale ZnO provides a large reactive surface area for radical interaction. Consequently, the superior antioxidant performance results from the synergistic effects of the ZnO core and phytochemical functionalization rather than either component alone. Similar mechanisms have been described for *Moringa*‐derived ZnO nanoparticles by Bhalla et al. (2023), Katyal et al. (2025), and Radulescu et al. (2023).

The inclusion of the extract‐only control therefore validates the central hypothesis of this study that phytochemical capping enhances the biological activity of green‐synthesized ZnO nanoparticles.

### 3.12 Storage Stability of Green‐Synthesized ZnO‐NPs

The long‐term stability of biosynthesized ZnO nanoparticles was evaluated over a 60‐day storage period under ambient dark conditions (Table 6) to assess their structural and functional integrity. The UV–visible absorption spectra demonstrated that the characteristic absorption peak initially observed at 365 nm remained nearly unchanged throughout the storage duration. A very slight blue shift from 365 to 363 nm was recorded after 60 days, indicating minimal alteration in particle size or surface state. The calculated band gap energy showed only a marginal change, from 3.40 eV at Day 0 to 3.42 eV after 60 days, indicating that the electronic properties of the nanoparticles remained largely stable during storage.

**TABLE 6:** Storage stability of green‐synthesized ZnO‐NPs during 60 days of storage at 25°C ± 2°C under dark conditions.

Storage time (days)	*λ*_max_ (nm)	Band gap (eV)	DPPH scavenging (%) at 100 μg/mL	% Retention of antioxidant activity
0	365	3.40	99.90 ± 0.42	100
15	365	3.40	98.85 ± 0.38	98.95
30	364	3.41	97.72 ± 0.44	97.82
45	364	3.41	96.64 ± 0.51	96.74
60	363	3.42	95.82 ± 0.57	95.92

*Note:* Values are expressed as mean ± SD (*n* = 3). No significant differences (*p* > 0.05) were observed in *λ*_max_ values during storage.

The negligible spectral shift suggests that the phytochemicals derived from *M. oleifera* leaf extract effectively acted as capping and stabilizing agents, preventing agglomeration and oxidative degradation during storage. Similar stabilization mechanisms have been reported in plant‐mediated ZnO nanoparticle systems, where surface‐bound phenolic and flavonoid compounds enhance colloidal stability and prevent structural deterioration (Bhalla et al. 2023; Radulescu et al. 2023).

The antioxidant activity exhibited high retention during storage. The DPPH radical scavenging efficiency decreased slightly from 99.90% at Day 0 to 95.82% after 60 days at 100 μg/mL concentration, corresponding to approximately 95.9% retention of activity. The gradual reduction may be attributed to minor surface rearrangements or partial desorption of loosely bound phytochemical molecules. However, the reduction was not statistically significant (*p* > 0.05), indicating excellent storage stability. The sustained antioxidant functionality demonstrates that the green synthesis approach imparts long‐term stability, highlighting the potential of these ZnO‐NPs for future food, pharmaceutical, and biomedical applications. However, further safety evaluations, including cytotoxicity and biocompatibility studies, are required before practical application.

### 3.13 Kinetic Analysis of DPPH Radical Scavenging Behavior

To gain deeper mechanistic insight into the antioxidant behavior of the biosynthesized ZnO nanoparticles, the time‐dependent DPPH radical scavenging data were analyzed using a pseudo‐first‐order kinetic model. The reduction in DPPH absorbance at 517 nm was monitored over 60 min, and the linearized plots of ln(*A*_*t*_/*A*_0_) versus time exhibited strong linear correlations (*R* ^2^ > 0.95), confirming that the radical scavenging reaction follows pseudo‐first‐order kinetics with respect to DPPH concentration. This behavior indicates that the rate of radical neutralization is directly proportional to the concentration of unreacted DPPH radicals remaining in the reaction medium (Table 7).

**TABLE 7:** Pseudo‐first‐order kinetic parameters of DPPH scavenging by ZnO nanoparticles.

Sample	Conc. (μg/mL)	*k* (min^−1^)	*R* ^2^	*t*_1/2_ (min)
Green ZnO‐NPs	100	0.082 ± 0.003^a^	0.978	8.45 ± 0.31^a^
Green ZnO‐NPs	50	0.057 ± 0.002^b^	0.969	12.16 ± 0.42^b^
Green ZnO‐NPs	25	0.039 ± 0.001^c^	0.961	17.77 ± 0.50^c^
AR‐grade ZnO	100	0.019 ± 0.001^d^	0.952	36.48 ± 1.12^d^

*Note:* Values represent mean ± standard deviation (*n* = 3). Different superscripts within a column indicate significant difference at *p* < 0.05.

The pseudo‐first‐order kinetic model was employed to compare the antioxidant reaction rates of biosynthesized ZnO‐NPs at representative low (25 μg/mL), intermediate (50 μg/mL), and high (100 μg/mL) concentrations, consistent with the concentration set reported in Table 7. These concentrations adequately illustrate the concentration‐dependent kinetic behavior of the nanoparticles and have been used to evaluate reaction rate constants and half‐life values in previous DPPH kinetic studies (Gulcin and Alwasel 2023; Katyal et al. 2025).

The calculated kinetic parameters revealed a pronounced enhancement in reaction rate for the green‐synthesized ZnO‐NPs compared to analytical‐grade (AR) ZnO. At a concentration of 100 μg/mL, the apparent rate constant (*k*) for the biosynthesized nanoparticles was 0.082 min^−1^, which was substantially higher than that of AR‐grade ZnO (0.019 min^−1^). Correspondingly, the half‐life (*t*_1/2_) of the DPPH radical was significantly shorter in the presence of green ZnO‐NPs (8.45 ± 0.31 min) than with AR‐grade ZnO (36.48 ± 1.12 min). These results demonstrate that the phytochemically synthesized nanoparticles possess markedly faster electron transfer kinetics and superior radical quenching efficiency.

The rapid decline in DPPH absorbance observed during the initial 30–40 min suggests the presence of abundant reactive surface sites capable of donating electrons or hydrogen atoms to stabilize free radicals. The enhanced kinetic behavior of the green‐synthesized ZnO‐NPs can be attributed to several synergistic factors. First, the nanoscale particle size (45–82 nm) provides a high surface area‐to‐volume ratio, facilitating increased interaction between nanoparticles and DPPH molecules. Second, the presence of surface‐bound phytochemicals particularly phenolics and flavonoids derived from *M. oleifera* leaf extract contributes directly to electron donation and radical stabilization. These bioorganic molecules act not only as reducing agents during nanoparticle synthesis but also as capping and stabilizing agents, preventing aggregation and maintaining accessible reactive surfaces. Similar mechanisms of enhanced redox activity in phyto‐mediated nanoparticles have been reported previously (Gul et al. 2025; Gulcin and Alwasel 2023; Katyal et al. 2025).

In contrast, AR‐grade ZnO exhibited considerably slower reaction kinetics, reflecting the absence of organic surface functionalization and limited intrinsic electron‐donating capacity. Without phytochemical capping molecules, the antioxidant activity of inorganic ZnO is primarily dependent on surface defects and intrinsic semiconductor properties, which appear insufficient to produce rapid radical neutralization. The clear kinetic distinction between the two samples highlights the critical role of plant‐derived bioactive compounds in modulating nanoparticle surface chemistry and enhancing antioxidant performance (Katyal et al. 2025; Gul et al. 2025).

Overall, the kinetic modeling results validate that the antioxidant action of the biosynthesized ZnO‐NPs is governed by pseudo‐first‐order reaction dynamics and is characterized by rapid, concentration‐dependent radical scavenging. The significantly higher rate constant and shorter half‐life confirm that phyto‐mediated synthesis not only improves total antioxidant capacity but also accelerates the electron transfer process, thereby producing functionally superior nanomaterials with promising potential for future biomedical and food‐related applications, subject to comprehensive safety and biocompatibility evaluation (Gulcin and Alwasel 2023).

## 4 Conclusion

This study demonstrated that the drying method significantly influences the phytochemical composition of *M. oleifera* leaves. Freeze‐drying preserved the highest levels of phenolics, flavonoids, vitamin C, and β‐carotene, making it the most suitable pretreatment for subsequent nanoparticle synthesis; the four drying methods were therefore compared only at the phytochemical‐retention stage, and ZnO nanoparticles were synthesized and characterized exclusively from the selected freeze‐dried extract, rather than from all four drying treatments. The resulting ZnO nanoparticles were successfully synthesized and comprehensively characterized by UV–Vis, FT‐IR, XRD, SEM, EDS, dynamic light scattering, and zeta potential analyses, confirming their crystalline structure, flower‐like morphology, nanoscale size (45–82 nm), and excellent colloidal stability. Compared with analytical‐grade ZnO, the biosynthesized nanoparticles exhibited superior antibacterial and antioxidant activities, faster pseudo‐first‐order radical scavenging kinetics, and excellent storage stability over 60 days, highlighting the synergistic role of phytochemical capping. These findings establish freeze‐drying as an effective pretreatment for producing stable and bioactive ZnO nanoparticles through an eco‐friendly synthesis approach. Future studies should investigate cytotoxicity, biocompatibility, and in vivo efficacy to facilitate their safe application in food, pharmaceutical, and biomedical fields.

## Author Contributions

**Tamanna Rahman:** investigation, methodology, data curation, formal analysis, writing – original draft. **Fahima Siddikey:** investigation, validation, formal analysis, data curation, writing – original draft. **Md. Hassan Bin Nabi:** methodology, formal analysis, software, validation, writing – review and editing. **Md Abu Rayhan:** methodology, software, data curation, visualization, validation. **Wahidu Zzaman:** conceptualization, formal analysis, validation, visualization, project administration, writing – review and editing, supervision, resources.

## Funding

The authors have nothing to report.

## Conflicts of Interest

The authors declare no conflicts of interest.

## Data Availability

The data that support the findings of this study are available from the corresponding author upon reasonable request.
